# Hydrothermal Embedding of Pd Single Atoms Into SnO_2_ for Efficient CO Oxidation

**DOI:** 10.1002/advs.75534

**Published:** 2026-05-05

**Authors:** Yingsheng An, Min Xiao, Mengyuan Zhang, Yao Lv, Yanwei Sun, Zhi Liu, Wenbo Pei, Hao Guo, Guangyan Xu, Yong Yan, Sheng Dai, Yunbo Yu, Hong He

**Affiliations:** ^1^ Laboratory of Atmospheric Environment and Pollution Control Research Center for Eco‐Environmental Sciences Chinese Academy of Sciences Beijing China; ^2^ University of Chinese Academy of Sciences Beijing China; ^3^ Ganjiang Innovation Academy Chinese Academy of Sciences Ganzhou China; ^4^ Key Laboratory for Advanced Materials, Feringa Nobel Prize Scientist Joint Research Center, School of Chemistry and Molecular Engineering East China University of Science and Technology Shanghai China; ^5^ College of Ecology and Environment Xinjiang University Urumqi China; ^6^ School of Chemical Engineering Technology Xinjiang University Urumqi China; ^7^ State Key Laboratory of Advanced Environmental Technology, Institute of Urban Environment Chinese Academy of Sciences Xiamen China

**Keywords:** CO oxidation, lattice incorporation, O_2_ activation, Mars‐van Krevelen Mechanism, single atom catalyst

## Abstract

This work employs a hydrothermal embedding strategy to fabricate a highly efficient and stable Pd/SnO_2_ catalyst for CO oxidation. With only 0.15 wt.% Pd, the hydrothermally treated catalyst (Pd/SnO_2_‐H) exhibits a reaction rate three times that of the fresh counterpart (Pd/SnO_2_‐F). Combined experimental and theoretical evidence reveals that hydrothermal treatment redistributes Pd species and partially incorporates Pd atoms into the SnO_2_ lattice, generating single‐atom Pd sites (Pd_1_). These lattice‐incorporated Pd_1_ sites strengthen the Pd‐SnO_2_ interaction, which weakens the Pd─O bond, thereby promoting the formation of oxygen vacancies and, more importantly, facilitating the subsequent rate‐determining O_2_ activation step. This enables a low‐barrier Mars–van Krevelen pathway, in contrast to the higher‐energy Langmuir–Hinshelwood route operating on PdO clusters in Pd/SnO_2_‐F, thereby explaining the high intrinsic activity of Pd/SnO_2_‐H. Furthermore, Pd lattice doping modulates the *d*‐electron distribution of Pd and weakens CO adsorption, effectively alleviating the CO poisoning typically observed on conventional Pd nanoparticles. Together, these results establish a new design principle for high‐performance single‐atom catalysts, demonstrating that lattice‑embedding of the active metal into a reducible oxide support can simultaneously enhance redox kinetics and suppress poisoning.

## Graphical abstract

By applying hydrothermal regulation to SnO_2_‐supported PdO, we achieve atomic dispersion of Pd into the SnO_2_ lattice, which activates the lattice oxygen neighboring Pd atoms to generate oxygen vacancies and facilitates subsequent O_2_ activation, thereby promoting CO oxidation to proceed via a low‐energy‐barrier MvK pathway while avoiding CO poisoning.

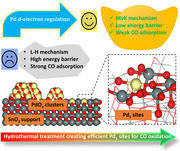

## 1 Introduction

Catalytic oxidation of carbon monoxide (CO) is critical for purifying vehicle exhaust, industrial flue gases, and feedstock streams for fuel cells [1, 2, 3]. Supported noble‐metal catalysts, especially those based on Pt and Pd, have been widely investigated for CO oxidation owing to their high activity [4, 5, 6, 7, 8, 9]. However, their commercialization remains hindered by susceptibility to CO poisoning and the high cost of noble metals [2, 3, 10]. Oxide‐supported single‐atom catalysts (SACs), first conceptualized by Zhang and co‐workers [11], offer a promising solution. In SACs, noble metals are dispersed as isolated sites with nearly 100% atomic efficiency on oxide supports, enabling a dramatic reduction of metal usage. Moreover, the positively charged metal centers typically found in oxide‐supported SACs bind CO more weakly than conventional metal nanoparticles, thereby suppressing CO poisoning [12].

Recently, single‐atom noble metals on reducible oxides, such as Pt_1_/CeO_2_, Pd_1_/CeO_2_, and Pd_1_/TiO_2_, have shown outstanding low‐temperature CO‐oxidation activity [13, 14, 15, 16]. This performance arises from a Mars–van Krevelen (MvK) pathway wherein the isolated metal atom cooperates with adjacent lattice oxygen of the support. The highly labile lattice oxygen in reducible oxides accelerates CO oxidation; its consumption generates oxygen vacancies that, in turn, promote O_2_ adsorption and activation. As a result, the kinetically limiting elementary step (often O_2_ activation or the subsequent CO‐to‐CO_2_ step) is substantially facilitated on these SACs [3, 17, 18].

In SACs, isolated metal atoms are stabilized via multiple interactions, including being trapped by vacancies or stepped edges [14, 19], anchored by surface hydroxyl groups [20, 21], and confined at the oxide–support interface [12, 22]. Interestingly, theoretical calculations have predicted that Pt_1_/CeO_2_ can be created by substituting a lattice Ce^4+^ cation with Pt, yielding a Pt^4+^‐O‐Ce^4+^ structure [23]. Adopting a similar substitution strategy, Pd_1_/CeO_2_ was successfully synthesized and shown to promote adjacent oxygen‐vacancy formation [24]. Nevertheless, CO oxidation on Pd_1_/CeO_2_ remains limited by O_2_ dissociation with a high energy barrier (1.52 eV). Notably, since oxygen vacancies in SnO_2_ have been reported to readily facilitate dissociative O_2_ adsorption [25, 26, 27], we hypothesize that lattice‐embedded, isolated Pd in SnO_2_ will lower the O_2_‐activation barrier and thereby deliver a highly active CO‐oxidation SAC.

Guided by these insights, we prepared a Pd single‐atom catalyst by hydrothermal aging of a fresh Pd/SnO_2_ (Pd/SnO_2_‐F) catalyst. The resulting Pd/SnO_2_‐H catalyst, with a low Pd loading of 0.15 wt.%, exhibited a CO oxidation reaction rate three times that of the fresh counterpart. As anticipated, lattice‐incorporated Pd_1_ sites facilitate the formation of oxygen vacancies and accelerate subsequent O_2_ activation, enabling a Mars–van Krevelen (MvK) pathway with a markedly lower barrier than the Langmuir–Hinshelwood route operative on PdO clusters in Pd/SnO_2_‐F. In parallel, Pd lattice doping redistributes Pd *d*‐electrons and reconfigures Pd─CO bonding, substantially weakening CO adsorption compared to Pd sites in PdO clusters. This reduced CO binding effectively alleviates the CO‐induced poisoning typically observed on conventional PdO nanoparticles.

## 2 Results and Discussion

### 2.1 Activity Evaluation and Reaction Kinetics

**FIGURE 1:**
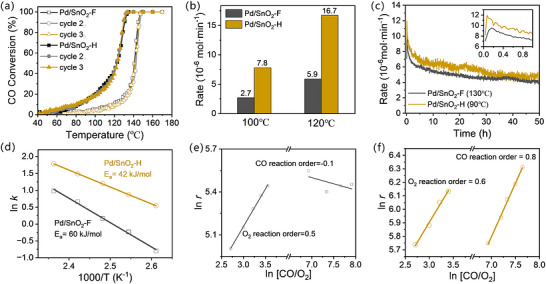
(a) CO light‐off curves during three rounds of successive cycles (40–160°C), (b) reaction rates at 100°C and 120°C, (c) long‐term stability curves (50 h), and (d) Arrhenius plots of Pd/ SnO_2_‐F and Pd/ SnO_2_‐H for CO oxidation. Reaction order test curves of (e) Pd/SnO_2_‐F (130°C) and (f) Pd/SnO_2_‐H (80°C) for CO oxidation.

The fresh and hydrothermally treated (at 750°C for 16 h in the presence of 10 vol% H_2_O and 3.5 % O_2_/N_2_) catalysts (denoted as Pd/SnO_2_‐F and Pd/SnO_2_‐H) have a similar Pd loading of ∼0.15 wt.%, as determined by inductively coupled plasma optical emission spectrometry (ICP‐OES) analysis (Table S1). CO oxidation was evaluated under kinetic control (Figure S1). Hydrothermal aging markedly enhanced catalytic activity, as evidenced by the ∼40°C decrease in the 10% conversion temperature (T_10_) for Pd/SnO_2_‐H (∼80°C) from ∼120°C for Pd/SnO_2_‐F (Figure 1a). Pd/SnO_2_‐H also showed robust cycle stability (Figure 1a) and delivered reaction rates nearly three times that of Pd/SnO_2_‐F at both 100 and 120°C (Figure 1b). The catalytic activity of Pd/SnO_2_‐H remains highly competitive against typical CO oxidation catalysts reported in the literature (Table S2). In long‐term stability tests (Figure 1c), both samples exhibited a short activation period followed by stable rates over 50 h. Notably, the stabilized reaction rate of Pd/SnO_2_‐H at 90°C matched that of Pd/SnO_2_‐F at 130°C, further confirming the significant enhancement of its low‐temperature activity.

Kinetic analyses elucidate the impact of hydrothermal treatment on intrinsic activity and mechanism. Arrhenius plots (Figure 1d) show a markedly lower apparent activation energy for Pd/SnO_2_‐H (42 kJ·mol^−1^) than for Pd/SnO_2_‐F (60 kJ·mol^−1^). Pd/SnO_2_‐F displays reaction orders of +0.5 for O_2_ and ‐0.1 for CO, consistent with a Langmuir–Hinshelwood (L–H) pathway in which strongly adsorbed CO inhibits turnover (Figure 1e). In contrast, Pd/SnO_2_‐H exhibits a positive CO reaction order of +0.8, indicating that CO participates via weak adsorption or gas‐phase reaction, in stark contrast to the strong CO binding typical of conventional PdO nanoparticles (Figure 1f) [10, 28]. Collectively, these results demonstrate that hydrothermal treatment generates intrinsically more active Pd─SnO_2_ interfacial sites and shifts the dominant CO‐oxidation pathway.

### 2.2 Structural Elucidation and Evolution

**FIGURE 2:**
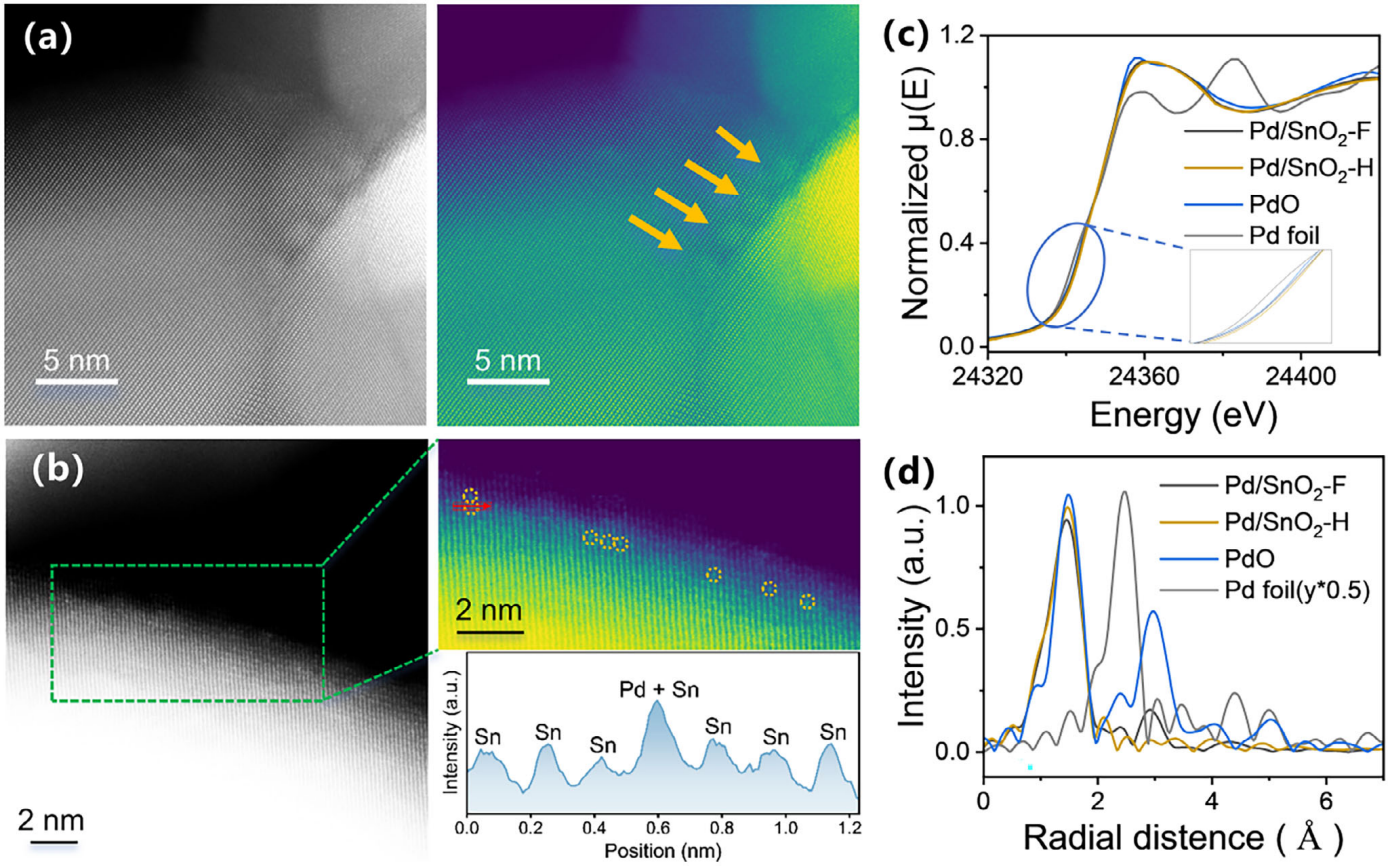
(a, b) Atomic‐resolution HAADF‐STEM images of (a) Pd/SnO_2_‐F and (b) Pd/SnO_2_‐H. The yellow arrows and circles highlight the Pd clusters and single atoms in the false‐colored image. The intensity profile is collected along the red dashed line region; (c) XANES spectra and (d) k^2^‐weighted FT‐EXAFS spectra.

To elucidate the structural evolution of hydrothermal treatment, comprehensive characterizations were undertaken. *X*‐ray diffraction (XRD) patterns of Pd/SnO_2_ (Figure S2) show only SnO_2_ diffraction peaks (JCPDS PDF# 00‐041‐1445, space group P42/mnm), with no distinct Pd‐related peaks, suggesting the high dispersion of Pd species. BET specific surface area and pore structure of the catalyst show only a negligible change after hydrothermal treatment (Table S1, Figures S3 and S4). Aberration‐corrected high‐angle annular dark field‐scanning transmission electron microscopy (HAADF‐STEM) images (Figure 2a,b) reveal that Pd exists as ∼2 nm clusters in Pd/SnO_2_‐F. In contrast, the aging treatment induces pronounced Pd redispersion, yielding numerous monodispersed Pd single atoms (Pd_1_), alongside a significant reduction in Pd cluster size. The intensity profile clearly demonstrates the enhanced *Z*‐contrast of the Pd_1_ site, while the observed Pd_1_ atoms are located exactly at the lattice fringes of SnO_2_. These changes indicate that hydrothermal treatment not only drives the redispersion of Pd species but also facilitates their incorporation into the SnO_2_ lattice. Further evidence supporting this structural transformation was obtained from energy‐dispersive X‐ray spectroscopy (EDS) mapping of Pd/SnO_2_‐H (Figure S5), which excludes the formation of large‐sized PdO particles during the redispersion process.

To gain deeper insight into the chemical states and coordination environments of Pd species, *X*‐ray absorption fine structure (XAFS) characterization was performed. As shown in Figure 2c, both catalysts display absorption edge energies and white‐line intensities in the *X*‐ray absorption near‐edge structure (XANES) spectra similar to those of PdO, indicating an average Pd valence state close to +2. In agreement with the XANES results, the Fourier‐transformed extended *X*‐ray absorption fine structure (FT‐EXAFS) spectra (Figure 2d and Figure S6) exhibit a first‐shell of Pd─O scattering at 1.45 Å, consistent with bulk PdO, with no detectable Pd─Pd scattering path. In the Pd/SnO_2_‐F sample, the second‐shell Pd─(O)─Pd scattering peak is substantially attenuated compared to that of PdO, consistent with the presence of highly dispersed, small‐sized PdO clusters. Notably, this second‐shell peak nearly disappears in the Pd/SnO_2_‐H sample, further verifying the enhanced dispersion of Pd species and the formation of isolated Pd_1_. Quantitative EXAFS fitting results (Table S4 and Figure S7–S9) reveal that the Pd/SnO_2_‐H catalyst exhibits a higher average Pd─O coordination number than Pd/SnO_2_‐F (increasing from 3.44 to 3.63) and a slightly elongated Pd─O bond length (from 2.01 to 2.03 Å). These differences indicate that hydrothermal treatment not only enhances Pd dispersion but also modifies its local coordination environment, consistent with the incorporation of Pd species into the SnO_2_ lattice.

**FIGURE 3:**
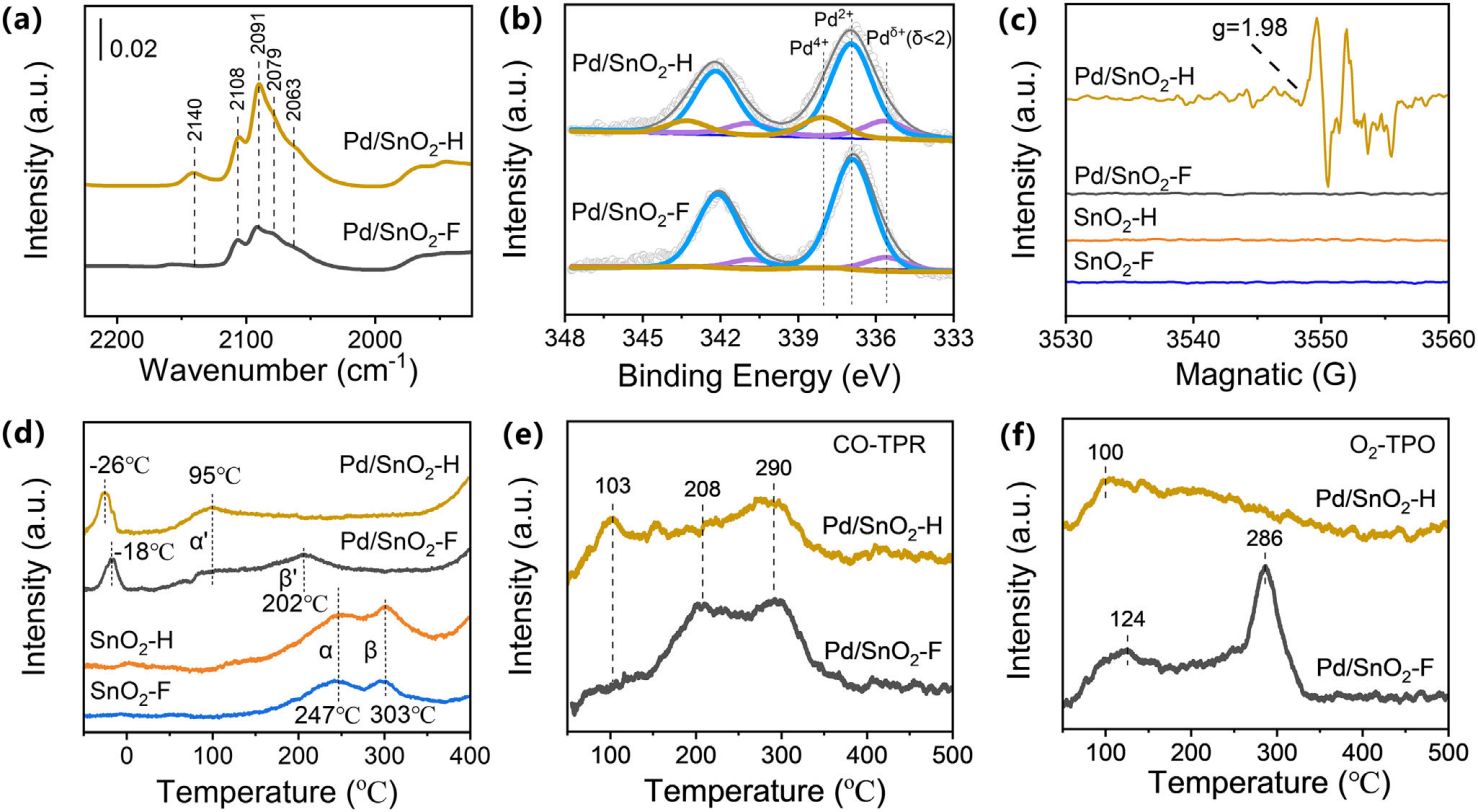
(a) CO adsorption DRIFTS, (b) Pd 3d XPS profiles for Pd/SnO_2_‐F and Pd/SnO_2_‐H, (c) EPR profiles and (d) H_2_‐TPR profiles for SnO_2_‐F, SnO_2_‐H, Pd/SnO_2_‐F, and Pd/SnO_2_‐H, (e) CO‐TPR, (f) O_2_‐TPO profiles for Pd/SnO_2_‐F and Pd/SnO_2_‐H.

The structural evolution of Pd sites following hydrothermal treatment was further corroborated by CO‐DRIFTS measurements (Figure 3a). After treatment, the integrated intensity of the linearly adsorbed CO bands (2050–2120 cm^−1^) [29, 30, 31] increases markedly, evidencing finer dispersion of Pd species and a reduced size of PdO clusters. Notably, a new peak appears at 2140 cm^−1^, which is attributed to CO adsorption on isolated Pd_1_ sites [10, 31]. These spectral changes provide additional evidence for the formation of atomically dispersed Pd species upon hydrothermal aging, accompanied by a corresponding evolution in the chemical state of Pd as revealed by X‐ray photoelectron spectroscopy (XPS) analysis (Figure 3b, Table S3). The Pd/SnO_2_‐H catalyst exhibits not only the typical features of Pd^2+^ (336.9 eV) [32] and Pd^δ+^ (δ < 2, 335.6 eV) [33], indicative of PdO_x_ clusters dominated by Pd^2+^, but also a distinct Pd^4+^ component at ∼338 eV [32, 33]. The presence of this Pd^4+^ species aligns well with the Sn‐substituted Pd dopants inferred from our HAADF‐STEM images. Taken together, these complementary spectroscopic results confirm that hydrothermal treatment not only promotes the redispersion of PdO clusters but also facilitates the partial incorporation of Pd atoms into the SnO_2_ lattice and generates isolated Pd_1_ sites. This atomic‐scale structural transformation triggers a distinct reaction pathway, as discussed in the following section.

### 2.3 Unraveling the CO Oxidation Pathway

The activation of O_2_ is pivotal for CO oxidation, often governing the overall reaction efficiency [3, 17, 18, 20]. Given the structural transformation identified above, we next investigated the impact of this atomic‐scale modification on oxygen activation and the subsequent CO oxidation pathway. Electron paramagnetic resonance (EPR) spectroscopy was employed to probe the formation of oxygen vacancies. As shown in Figure 3c, a distinct signal at g = 1.98 appears exclusively for Pd/SnO_2_‐H, characteristic of oxygen vacancies in SnO_2_ [34]. In contrast, no such signal is observed for Pd/SnO_2_‐F, SnO_2_‐F, and SnO_2_‐H, indicating that the formation of oxygen vacancies is intimately linked to the presence of lattice‐incorporated Pd_1_ sites. Complementary evidence was obtained from O 1s XPS analysis (Figure S10). Quantitative peak fitting results reveal that hydrothermal treatment decreases the proportion of lattice oxygen (O_I_), while increasing the content of defect‐related oxygen species (O_II_) from 15% to 18% [35]. This shift further corroborates the enhanced formation of oxygen defects induced by Pd incorporation, suggesting a modified metal‐support interaction.

The metal–support interaction was further probed by H_2_ temperature‐programmed reduction (H_2_‐TPR). As displayed in Figure 3d, the pristine SnO_2_ support exhibits two reduction peaks at 247°C and 303°C, assigned to surface adsorbed oxygen (*α*) and surface lattice oxygen (*β*), respectively [36]. Upon Pd loading, both peaks shift to lower temperatures, to 95°C and 202°C, suggesting a strong Pd‐SnO_2_ interaction that facilitates oxygen activation. Surprisingly, the reduction peak corresponding to surface lattice oxygen of SnO_2_ disappears in the Pd/SnO_2_‐H sample. This distinct behavior indicates a substantially altered metal‐support interaction, wherein the lattice‐incorporated Pd_1_ sites perturb the local coordination environment of SnO_2_ and thereby modify the activation pathway of surface lattice oxygen. Consistent with this change, the reduction feature associated with PdO species in Pd/SnO_2_‐H appears at a lower temperature than that of the fresh counterpart (−26°C vs −18°C) and exhibits a larger peak area. This shift, aligning with the increased Pd─O bond length and coordination number revealed by EXAFS, confirms that lattice‐embedded Pd_1_ sites induce a more intimate metal‐support interaction, thereby generating more reactive Pd─O bonds.

The reactive nature of the Pd─O bonds in the hydrothermally treated sample was also supported by O_2_ temperature‐programmed desorption (O_2_‐TPD) measurements (Figure S11). In contrast to Pd/SnO_2_‐F, which exhibits only one dominant desorption peak at approximately 450°C, Pd/SnO_2_‐H displays an additional low‐temperature O_2_ desorption peak at around 400°C, indicating the presence of oxygen species associated with weakened Pd─O bonds and enhanced reactivity. The involvement of lattice oxygen in the CO oxidation process was directly probed by CO temperature‐programmed reduction (CO‐TPR, Figure 3e). Pd/SnO_2_‐H exhibits a reduction onset at a low temperature of ∼50°C, which coincides well with its light‐off temperature, implying that lattice oxygen adjacent to Pd_1_ sites can be readily consumed by CO even under low‐temperature conditions. Complementary evidence for the reversible participation of lattice oxygen was obtained from O_2_ temperature‐programmed oxidation measurements (O_2_‐TPO, Figure 3f). The pre‐reduced Pd species in Pd/SnO_2_‐H are readily re‐oxidized at low temperatures, indicating rapid replenishment of oxygen vacancies generated during CO oxidation under oxygen‐rich conditions. Such facile reoxidation is essential for stabilizing the atomically dispersed Pd_1_ sites, as prompt restoration of lattice oxygen suppresses the aggregation of reduced Pd species. Consistently, after a 50 h long‐term stability test (Figure S12), abundant isolated Pd atoms remain uniformly dispersed along the lattice fringes of SnO_2_, underscoring the excellent structural stability of the Pd/SnO_2_‐H catalyst. Collectively, these results support a low‐temperature MvK pathway on Pd/SnO_2_‐H in which lattice oxygen adjacent to Pd_1_ sites is activated, participates readily in CO oxidation, and is efficiently replenished, accounting for its enhanced intrinsic activity and excellent stability.

**FIGURE 4:**
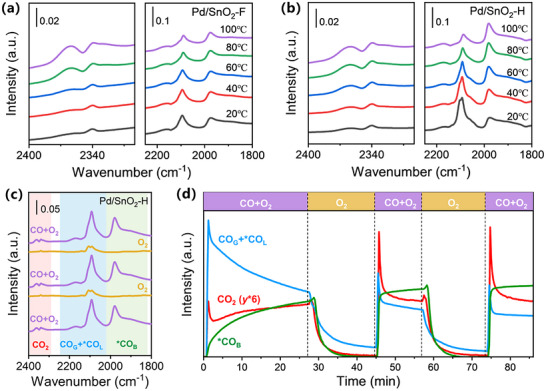
Dynamic changes of in situ DRIFTS spectra during the temperature‐ramping process under the CO oxidation conditions for (a) Pd/SnO_2_‐F and (b) Pd/SnO_2_‐H. (c) In situ DRIFTS spectra of Pd/SnO_2_‐H during the alternating feeds of CO+O_2_ and O_2_ at 60°C, and (d) the time‐dependent curves of the integrated areas corresponding to CO_G_+^*^CO_L_, CO_2_, and ^*^CO_B_ species.

Conventional Pd catalysts are susceptible to strong CO adsorption and poisoning [10, 28]. By contrast, the Pd/SnO_2_‐H catalyst exhibits a distinct kinetic behavior, wherein CO reacts predominantly from the gas phase or in a weakly adsorbed state. The improved low‐temperature activity is further revealed by in situ diffuse reflectance infrared Fourier‐transform spectroscopy (DRIFTS) under CO oxidation conditions. As shown in Figure 4b, CO_2_ doublet peaks (2300–2400 cm^−1^) [37] emerge at 60°C on Pd/SnO_2_‐H, whereas on Pd/SnO_2_‐F, these peaks appear at a relatively higher temperature of ∼80°C (Figure 4a). This lower onset temperature for CO_2_ formation aligns with the enhanced activity of the hydrothermally treated catalyst.

To probe whether adsorbed CO participates directly at low temperatures, CO‐TPD and CO‐TPO profiles were obtained for Pd/SnO_2_‐H after CO pre‐adsorption (Figure S13). Under both oxygen‐free (CO‐TPD) and oxygen‐containing (CO‐TPO) conditions, the linear─CO band decays monotonically with temperature and vanishes near ∼150°C, indicating that strongly bound CO is not the dominant reactive intermediate in the low‐temperature regime. These observations, together with positive CO reaction order from kinetic analysis, support a pathway wherein weakly adsorbed or gaseous CO reacts with activated lattice oxygen on Pd_1_─SnO_2_ sites, consistent with the MvK framework established above.

To further verify this, in situ DRIFTS was performed at 60°C under alternating CO + O_2_ and O_2_ feeds (Figure 4c). Peak evolutions were quantified (Figure 4d) after assigning the following bands: 2300–2400 cm^−1^ for CO_2_, 2020–2250 cm^−1^ for both gaseous CO (CO_G_) [13] and linearly adsorbed CO (^*^CO_L_), and 1850–2020 cm^−1^ for bridge‐adsorbed CO (^*^CO_B_) [38]. Following the second CO + O_2_ pulse, the spectra reached a steady state. The CO_2_ signal tracked closely with the combined intensity of CO_G_ + ^*^CO_L_, whereas no correlation was observed with ^*^CO_B_. Specifically, upon each introduction of the CO + O_2_ feed, the decline in CO_2_ peak intensity was consistently accompanied by an elevation in the *CO_B_ peak intensity. This result indicates that the formation of the *CO_B_ species is associated with reduced CO_2_ production and does not directly participate in the CO oxidation. Upon switching off the CO feed, CO_2_ intensity decayed to baseline while ^*^CO_L_ persisted, consistent with CO‐TPO results (Figure S13) and indicating that linearly adsorbed CO is not the reactive intermediate at low temperatures. Consequently, these data confirm that gaseous (or very weakly adsorbed) CO correlates most strongly with CO_2_ formation and directly participates in CO oxidation over Pd/SnO_2_‐H.

Previous DRIFTS studies on Pd/CeO_2_ assigned the peak at ca. 2140 cm^−1^ to CO bound to isolated Pd^2+^ species [39], whereas lower‐frequency bands were attributed to Pd species of lower oxidation state or larger particle size [39, 40]. In our case, the lattice‐embedded Pd_1_ sites in Pd/SnO_2_‐H are mainly present as Pd^4+^ according to XPS analysis. If CO adsorbs on these sites, the characteristic peak would be expected at an even higher frequency. This indicates that the bands between 2108 and 2063 cm^−1,^ assignable to ^*^CO_L_ species observed on Pd/SnO_2_‐H (Figure 3a), mainly originate from PdO clusters rather than Pd_1_ sites. Hydrothermal treatment reduces the size of PdO clusters, increases the number of accessible adsorption sites, and thus enhances the *CO_L_ signals. A weak band at 2140 cm^−1^ appeared only after CO exposure, likely due to partial reduction of trace Pd^4+^ to Pd^2+^ [39, 40].

### 2.4 DFT Insights Into the Reaction Mechanism

**FIGURE 5:**
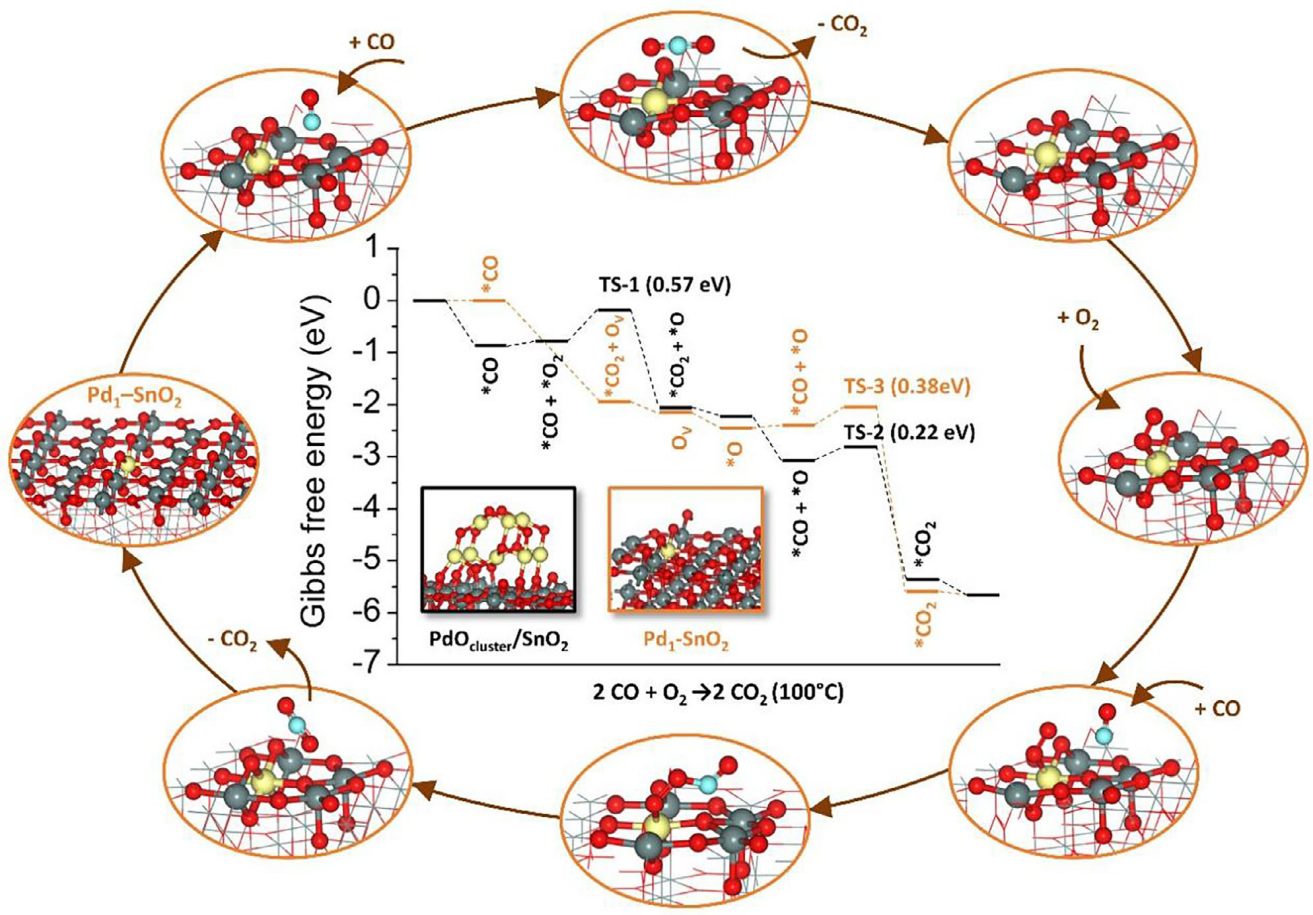
DFT‐calculated Gibbs free energy profiles of CO oxidation over PdO_cluster_/SnO_2_ and Pd_1_‐SnO_2_ models. The Gibbs free energy values were calculated using a temperature of 100 °C.

To gain atomic‐level insight into the distinct catalytic behavior and reaction pathway observed experimentally, DFT calculations were performed at different Pd sites. Two models were constructed on the thermodynamically stable SnO_2_(110) surface: a Pd single‐atom dopant (Pd_1_‐SnO_2_, Figure S14) and a supported PdO cluster (PdO_cluster_/SnO_2_, Figure S15). The Pd_1_‐SnO_2_ model shows a significantly higher Pd coordination number than the average Pd coordination number in the PdO_cluster_/SnO_2_ model (6.00 vs. 3.25), which is consistent with the EXAFS fitting results showing a higher Pd─O coordination number for Pd/SnO_2_‐H (3.63) than for Pd/SnO_2_‐F (3.44). Similarly, the average Pd─O bond length in the Pd_1_‐SnO_2_ model is longer than in the PdO_cluster_/SnO_2_ model (2.07 vs 1.97 Å), in line with the bond elongation detected by EXAFS. In addition, Bader charge analysis reveals that the average charge of Pd atoms in the PdO_cluster_/SnO_2_ model is 0.85, close to that of Pd in the primitive cell of PdO (0.92). In contrast, the single‐atom Pd_1_ in the Pd_1_ ‐ SnO_2_ model exhibits a substantially higher Bader charge of 1.38, indicative of a higher oxidation state. This result is in good agreement with the Pd 3*d* XPS results, which show a distinct Pd^4+^ component for Pd/SnO_2_‐H. Complete reaction profiles were computed for both (Figure 5; Figures S16 and S17). In agreement with the experimental findings, CO oxidation on Pd_1_‐SnO_2_ proceeds via a MVK pathway, wherein lattice oxygen adjacent to the Pd_1_ site participates directly. In contrast, the PdO_cluster_/SnO_2_ model follows an L–H mechanism involving co‐adsorbed CO and O_2_.

For the Pd_1_‐SnO_2_ model, the CO adsorption energy (ΔG at 100°C) is close to 0 eV, indicating that CO reacts from the gas phase or a weakly adsorbed state, consistent with kinetics and in situ DRIFTS results. Notably, the elementary step *CO + Pd_1_–O → *CO_2_ + Pd_1_–O_V_ proceeds without any energy barrier, indicating that Pd_1_ activates lattice oxygen and probably drives a MvK pathway. These findings further corroborate the EPR‐detected oxygen vacancies and the low‐temperature CO‐TPR reduction feature observed for Pd/SnO_2_‐H. On the Pd_1_‐SnO_2_ model, the rate‐determining step (RDS) for CO oxidation is the activation of O_2_ at the oxygen vacancy, with an energy barrier of 0.38 eV. This behavior is analogous to that of isolated Pd_1_ sites on CeO_2_, yet the corresponding barrier on Pd_1_‐SnO_2_ is markedly lower than the reported value of 1.52 eV for Pd_1_‐CeO_2_ [24]. Thus, Pd doping into SnO_2_ not only promotes the formation of oxygen vacancies but, more importantly, facilitates O_2_ activation at these vacancies. The overall barrier on Pd_1_‐SnO_2_ (0.38 eV) is also substantially lower than on PdO_cluster_/SnO_2_ (0.57 eV), aligning with the lower apparent E_a_ and explaining the superior activity of hydrothermally aged Pd/SnO_2_‐H.

To further substantiate the essential role of Pd_1_ lattice doping in SnO_2_, we also computed two CO‐oxidation pathways on a Pd_1_/SnO_2_ model where the isolated Pd atom resides on the SnO_2_(110) surface rather than being incorporated into the lattice. As shown in Figure S18‐ S20, both pathways exhibit high barriers (1.21 eV for Path 1 and 1.25 eV for Path 2), underscoring that lattice incorporation of Pd_1_, rather than mere surface anchoring, is critical for generating oxygen vacancies and enabling efficient O_2_ activation.

**FIGURE 6:**
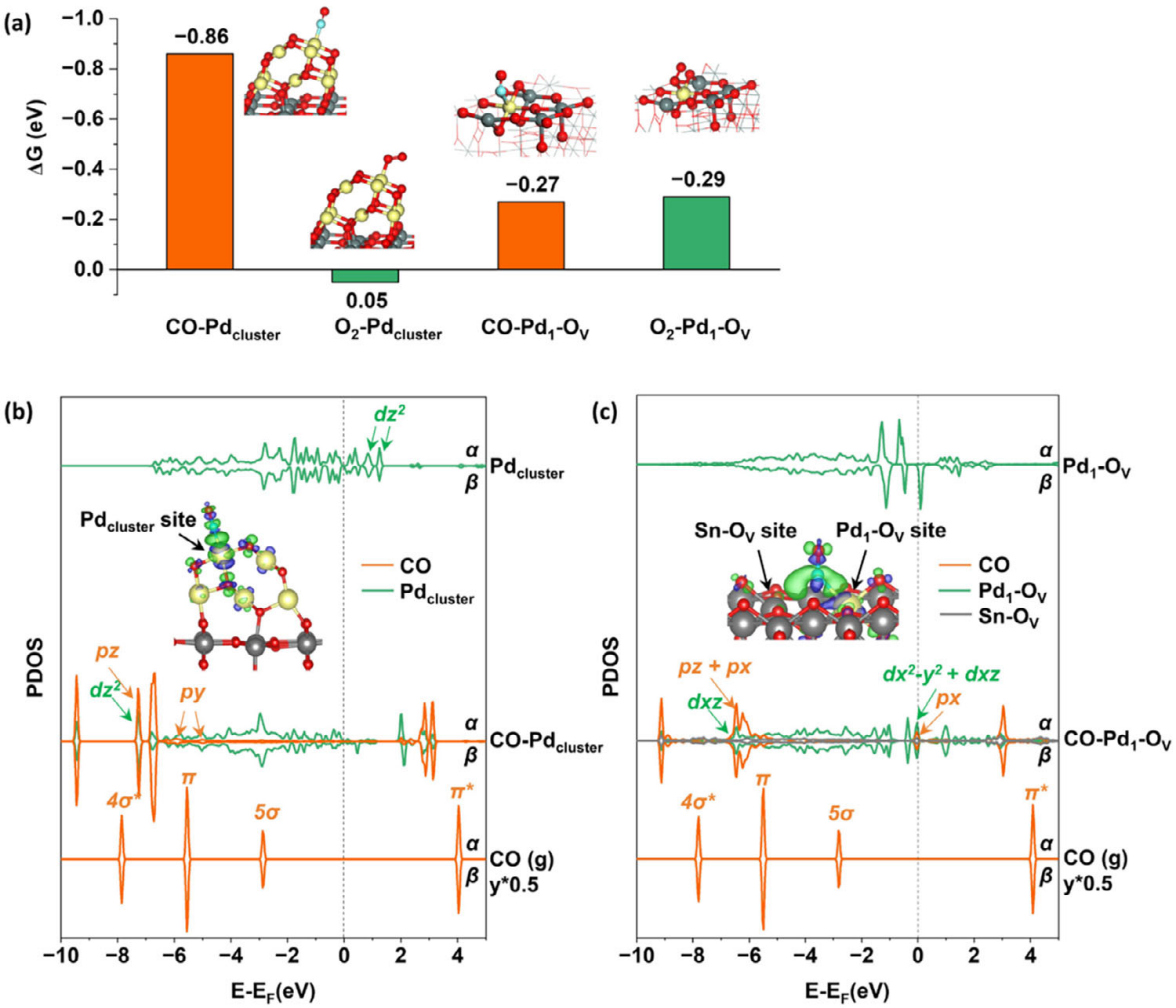
(a) The adsorption energy of CO and O_2_ on the Pd_cluster_ site of the PdO_cluster_/SnO_2_ model and the Pd_1_‐O_V_ site of the Pd_1_‐O_V_‐SnO_2_ model. The Gibbs free energy values were calculated using a temperature of 100°C. (b) PDOS of the Pd_cluster_ site on the PdO_cluster_/SnO_2_ model, CO gas molecule, and their interaction within CO adsorbed on the Pd_cluster_ site. (c) PDOS of the Pd_1_‐O_V_ site on the Pd_1_‐O_V_‐SnO_2_ model, the CO gas molecule, and their interaction within CO adsorbed on the Pd_1_‐O_V_ site. The insets in (b) and (c) are the differential charge density profiles (blue stands for holes and green for electrons) for the CO‐adsorbed Pd_cluster_ site and Pd_1_‐O_V_ site, respectively.

Beyond differences in mechanism and intrinsic activity, we examined competitive CO and O_2_ adsorption on two sites: the Pd cluster site in the PdO_cluster_/SnO_2_ model and the Pd_1_‐O_V_ site in the Pd_1_‐SnO_2_ model bearing a neighboring oxygen vacancy (Pd_1_‐O_V_‐SnO_2_, Figure 6a). On the Pd cluster, CO adsorbs much more strongly than O_2_ (ΔG_ads_ (CO) = −0.86 eV vs ΔG_ads_ (O_2_) = +0.05 eV), consistent with the negative CO reaction order observed for Pd/SnO_2_‐F. In contrast, on Pd_1_‐O_V_, CO adsorption is markedly weakened, while O_2_ adsorption is strengthened (ΔG_ads_ (CO) = −0.27 eV vs ΔG_ads_ (O_2_) = −0.29 eV), indicating that Pd_1_‐O_V_ sites both mitigate CO poisoning and promote O_2_ adsorption and activation.

Given this distinct CO adsorption behavior, we analyzed the electronic structure via projected density of states (PDOS) and differential charge density. For CO on the PdO_cluster_/SnO_2_ model (Figure 6b), the 5*σ* orbital of CO donates into the Pd *d* orbital (dominated by *dz* ^2^), forming low‐energy bonding orbitals and high‐energy unoccupied antibonding orbitals, thereby lowering the total energy. Furthermore, partial *d*‐electrons of Pd back‐donate to the π* orbital of CO, forming a feedback *π*‐bond that further reinforces CO adsorption. This is evident from the appearance of the small CO *py* peaks below the Fermi level (attributed to back‐donation) and the splitting of the π* orbital.

For CO adsorbed on the Pd_1_‐O_V_‐SnO_2_ model (Figure 6c), the dominant interaction is localized at the Pd_1_‐O_V_ site. Before adsorption, the Pd *d*‐band exhibits few low‐lying vacant states and is notably depleted in the *dz* ^2^ component, a consequence of the high coordination/oxidation state of lattice‐doped Pd. This electronic structure suppresses strong σ donation from CO into *dz* ^2^, thereby weakening *σ*‐*d* coordination. Differential charge‐density maps indicate a partial rehybridization of the CO carbon toward *sp* ^2^‐like character. This change is also reflected in the PDOS, where the *px* and *pz* hybrid orbitals of CO engage in covalent bonding with the *d* orbitals of Pd. Collectively, single‐atom Pd doping tailors the d‐electron distribution and bonding configuration to attenuate CO binding, mitigating the CO‐poisoning propensity typical of conventional Pd sites.

High‐temperature Ostwald ripening is a well‐known degradation pathway for heterogeneous catalysts. However, emerging research reveals that carefully controlled thermal or hydrothermal treatments can be employed to redirect metal mobility, trapping atoms at surface defects or step sites of reducible oxides such as CeO_2_, thereby constructing highly active single‐atom catalysts. For instance, physically mixing ceria with Pt/La‐Al_2_O_3_ followed by calcination in air at 800°C drives Pt migration onto ceria step sites, producing atomically dispersed Pt with excellent CO‐oxidation activity and stability, a step‐site trapping mechanism corroborated by DFT calculations [14, 41]. Similarly, hydrothermal treatment at 750°C, regardless of oxygen presence, preserves Pt^2+^ as an isolated species on CeO_2_ and activates surface lattice oxygen, enabling CO oxidation below 100°C [13]. Oxidative treatment (O_2_, 400°C) of Cr_2_O_3_/CeO_2_ likewise redistributes Cr_2_O_3_ nanoparticles into vacancy‐trapped Cr^n+^ single atoms (Cr_1_/CeO_2_) [42]. In contrast, this work introduces a distinct hydrothermal embedding strategy that directly incorporates Pd atoms into the lattice of SnO_2_. This approach not only creates exceptionally stable Pd_1_ sites but also utilizes the resulting lattice ‑ embedded structure to simultaneously facilitate O_2_ activation and weaken CO adsorption, thereby addressing two key limitations in low‐temperature CO oxidation.

## 3 Conclusion

We here demonstrate that hydrothermal pretreatment markedly enhances the CO oxidation performance of Pd/SnO_2_. This treatment redistributes Pd clusters and incorporates a fraction of Pd atoms into the SnO_2_ lattice, forming highly active Pd_1_ sites. Lattice doping tunes the Pd *d*‐band, thereby suppressing strong CO adsorption on the hydrothermally pretreated Pd/SnO_2_ (Pd/SnO_2_‐H) surface. On the Pd/SnO_2_‐H, moreover, the Pd_1_‐SnO_2_ interaction significantly weakens the Pd─O bond; the elementary reaction step between CO and lattice oxygen to form CO_2_ proceeds without an energy barrier, which generates oxygen vacancies in the vicinity of the Pd_1_ sites. At these vacancies, the subsequent O_2_ activation — the rate‐determining step for CO oxidation — exhibits a substantially lower barrier compared to that on PdO clusters in Pd/SnO_2_. This enables a Mars–van Krevelen (MvK) pathway, involving the reaction of lattice oxygen with gaseous CO, followed by O_2_ activation at the oxygen vacancies of Pd/SnO_2_‐H. As a result, Pd/SnO_2_‐H achieves a light‐off temperature (T_10_) as low as ∼80°C, underscoring its strong potential for practical application. These findings establish a distinct hydrothermal embedding strategy for incorporating noble metals into reducible oxides, offering a promising approach for designing next‐generation single‐atom catalysts.

## Conflicts of Interest

The authors declare no conflicts of interest.

## Supporting information




**Supporting File**: advs75534‐sup‐0001‐SuppMat.docx.

## Data Availability

The data that support the findings of this study are available from the corresponding author upon reasonable request.
