# Supplementary material for: Hydrothermal Embedding of Pd Single Atoms Into SnO2 for Efficient CO Oxidation
Source: Adv Sci (Weinh). 2026 May 5;13(42):e75534. doi: 10.1002/advs.75534 (PMC13335604; doi:10.1002/advs.75534)
Supplement: Supplementary file 1 — Supporting File: advs75534‐sup‐0001‐SuppMat.docx. [file ADVS-13-e75534-s001.docx]

Supporting Information

Hydrothermal Embedding of Pd Single Atoms into SnO_2_ for Efficient CO Oxidation

Yingsheng An^[a, b]^, Min Xiao^[c]^, Mengyuan Zhang^[a, b]^, Yao Lv^[d]^, Yanwei Sun^[a, b]^, Zhi Liu*^[a, b]^, Wenbo Pei^[f]^, Hao Guo^[g]^, Guangyan Xu^[a, b]^, Yong Yan*^[c]^, Sheng Dai*^[d]^, Yunbo Yu*^[a, b, c]^, Hong He^[a, b, e]^

[a] Y. An, M. Zhang, Y. Sun, Z. Liu, G. Xu, Y. Yu, H. He
Laboratory of Atmospheric Environment and Pollution Control, Research Center for Eco-Environmental Sciences, Chinese Academy of Sciences, Beijing 100085, China
*E-mail: zhiliu@rcees.ac.cn (Z. Liu); ybyu@rcees.ac.cn (Y. Yu)

[b] Y. An, M. Zhang, Y. Sun, Z. Liu, G. Xu, Y. Yu, H. He
University of Chinese Academy of Sciences, Beijing 100049, China

[c] M. Xiao, Y. Yan, Y. Yu
Ganjiang Innovation Academy, Chinese Academy of Sciences, Ganzhou 341119, China
*E-mail: yyan@gia.cas.cn (Y. Yan)

[d] Y. Lv, S. Dai
Key Laboratory for Advanced Materials, Feringa Nobel Prize Scientist Joint Research Center, School of Chemistry and Molecular Engineering, East China University of Science and Technology, Shanghai, China
*E-mail: shengdai@ecust.edu.cn (S. Dai)

[e] H. He
State Key Laboratory of Advanced Environmental Technology, Institute of Urban Environment, Chinese Academy of Sciences, Xiamen 361021, China

[f] W. Pei
College of Ecology and Environment, Xinjiang University, Urumqi, Xinjiang 830017, China

[g] H. Guo
School of Chemical Engineering Technology, Xinjiang University, Urumqi, Xinjiang 830017, China

***Corresponding Authors**

E-mail: zhiliu@rcees.ac.cn (Z. Liu); yyan@gia.cas.cn (Y. Yan); shengdai@ecust.edu.cn (S. Dai); ybyu@rcees.ac.cn (Y. Yu)

**The PDF file includes:**

Experimental Procedures

Supplementary Figure S1 to S20

Supplementary Table S1 to S4

Supplementary References

**Experimental Procedures**

Methods

**Synthesis of Pd/SnO_2_-F.** The fresh Pd/SnO_2_ catalyst (Pd/SnO_2_-F) was prepared by wet impregnation. SnO_2_ powder (10 g; 50–70 nm, Innochem) was dispersed in an aqueous Pd(NO_3_)_2_·2H_2_O solution (0.047 mol/L, 40 mL; Sigma-Aldrich, 99.995%) and stirred at room temperature for 8 h in a sealed vessel. The suspension was centrifuged, and the solid was washed with deionized water and recentrifuged three times. The product was dried at 80 °C for 2 h and calcined in air at 550 °C for 2 h. Inductively coupled plasma–optical emission spectrometry (ICP-OES) confirmed a Pd loading of 0.15 wt%.

**Synthesis of Pd/SnO_2_-H.** The hydrothermally aged catalyst (Pd/SnO_2_-H) was obtained by treating the fresh Pd/SnO_2_ in a flowing gas mixture of 10 vol% H_2_O and 3.5% O_2_ in N_2_ balance at 750 °C for 16 h. After cooling to room temperature, the solid was collected. ICP-OES analysis confirmed that the Pd loading remained 0.15 wt%.

**Characterization.** *X*-ray diffraction (XRD) was performed on a Bruker D8 Advance diffractometer using Cu Kα radiation (λ = 0.15406 nm). Patterns were collected over 2θ = 20–80° with a step size of 0.02°.

Aberration-corrected HAADF-STEM (AC-HAADF-STEM) was conducted on a Thermo Fisher Themis Z (300 kV, dual aberration correctors). Samples were dispersed in ethanol and drop-cast onto Au grids.

Energy-dispersive X-ray spectroscopy (EDS) mapping were performed using an electron microscope (JEM-2100F). Prior to measurement, the sample was thoroughly ground with a mortar, dispersed in ethanol, and then dropped onto a copper grid.

*X*-ray photoelectron spectroscopy (XPS) was acquired on a Thermo Scientific ESCALAB 250Xi with Al Kα radiation (hν = 1486.6 eV; 500 μm spot). Spectra were calibrated to the C 1s peak at 284.8 eV.

*X* -ray absorption fine structure (XAFS) measurements were performed at beamline BL13SSW of the Shanghai Synchrotron Radiation Facility using a Si(311) double-crystal monochromator and ionization chambers filled with Ar/N_2_. Pd K-edge (24,350 eV) XAS spectra were collected in fluorescence mode. EXAFS data were processed with Athena; k^2^-weighted Fourier transforms were used for analysis^[1]^.

N_2_ adsorption–desorption isotherms were recorded on a Micromeritics ASAP 2460. Specific surface areas were calculated by the BET method; pore volume and average pore diameter were obtained via the BJH model.

Palladium loadings were quantified by ICP-OES (Thermo Fisher iCAP 7400, USA).

H_2_-temperature-programmed reduction was performed on an AutoChem II 2920 (Micromeritics) equipped with a thermal conductivity detector (TCD). Each sample (100 mg) was pre-oxidized in 10 vol% O_2_/He (50 mL/min) at 400 °C for 1 h, then cooled to −50 °C under Ar. Reduction was conducted from −50 °C to 400 °C at 10 °C min^-1^ in 10 vol% H_2_/Ar (50 mL/min), and TCD signals were recorded continuously.

CO-temperature-programmed reduction was carried out on the same instrument (TCD). Samples were pretreated in 10 vol% O_2_/He (50 mL/min) at 400 °C for 1 h, cooled to room temperature under He, and then heated from ambient to 500 °C at 10 °C/min in 10 vol% CO/He (50 mL/min).

O_2_-temperature-programmed oxidation was also conducted on the AutoChem II 2920 (TCD). Samples (100 mg) were first exposed to 10 vol% H_2_/Ar (50 mL/min) at room temperature for 30 min. The gas was then switched to 2 vol% O_2_/He (30 mL/min) and held for 90 min before heating to 500 °C at 10 °C/min, followed by cooling. Oxygen uptake/release was monitored by the TCD.

O_2_-temperature-programmed desorption (O_2_-TPD) measurements were also performed on an AutoChem II 2920 instrument equipped with a thermal conductivity detector (TCD). For each measurement, 100 mg of sample was first treated in a 10 % O_2_/He (50 mL/min) at 600 °C for 60 min. The sample was then cooled down to room temperature, followed by purging with pure He (50 mL/min) for 60 min to remove physically adsorbed oxygen. Subsequently, the sample was heated to 600 °C at a rate of 10 °C/min, and the oxygen uptake and release during the process were monitored by the TCD detector.

Electron paramagnetic resonance (EPR) spectra were collected on a BRUKER EMX spectrometer. Approximately 100 mg of catalyst was loaded into a quartz EPR tube and flame-sealed prior to measurement.

*In-situ* diffuse reflectance FTIR spectroscopy was performed on a Bruker Vertex 80v equipped with a DRIFTS cell. Before each run, catalysts were pretreated at 400 °C for 2 h under 40 mL/min N_2_ and 10 mL/min O_2_. Subsequent experimental procedures were performed as follows: For the TPD experiment, a gas mixture containing 1000 ppm CO (balanced with N_2_) with a total flow rate of 50 ml/min was introduced to the pretreated catalyst at 20 °C. After 1 h of adsorption equilibrium, the CO supply was terminated, and temperature-programmed desorption was carried out at a heating rate of 10 °C/min over the temperature range of 20-200 °C; For the TPO experiment, a gas mixture containing 3.5% O_2_ and 1000 ppm CO (balanced with N_2_) with a total flow rate of 50 ml/min was introduced to the pretreated catalyst at 20 °C. After 1 h of adsorption, the CO supply was terminated, and temperature-programmed desorption was carried out at a heating rate of 10 °C/min over the temperature range of 20-200 °C; For the CO alternating feeds oxidation experiment, the reaction temperature was maintained constant at 60 °C. A gas mixture containing 3.5% O_2_ and 1000 ppm CO (balanced with N_2_) with a total flow rate of 50 ml/min was introduced to the pretreated catalyst. Once steady-state conditions were achieved, the CO supply was cut off until the system re-established stability. CO was then reintroduced, and this alternating cycle was repeated three times.

**Catalytic oxidation of CO.** Catalytic performance was evaluated in a fixed-bed quartz tubular reactor at 1 atm. In each run, 0.20 g of catalyst (40–60 mesh) was mixed with quartz sand (mass ratio catalyst:sand = 1: 2) and placed in the isothermal zone. Light-off curves were collected under a feed of 1000 ppm CO, 3.5 % O_2_, balance N_2_, at a high WHSV of 300000 ml·h^-1^·g_cat_^-1^. The bed was heated at 1 °C/min^-1^ to the target temperature. CO concentration was monitored online by FT-IR (Nicolet iS10, Thermo Fisher). CO conversion (X_CO_) and reaction rate (r) were calculated as follows (r evaluated at X_CO_ = 5–20 %) :

$X_{CO}=\left(1-\frac{\left[CO\right]_{out}}{\left[CO\right]_{in}}\right)\times100\%$ (1)

$r=\frac{L\times\left[CO\right]_{in}\times X_{CO}}{V_{m}}$ (2)

where [CO]_i_*_n_* and [CO]_out_ were the CO concentrations (ppm) at the inlet and outlet of the reactor, respectively; *L* represents the total flow rate (mL/min); *V_m_* represents the molar volume of gas.

**Theoretical calculations.** Spin-polarized DFT calculations were performed with VASP^[2-3]^ using the PAW method and a plane-wave cutoff of 400 eV^[4-5]^. Exchange–correlation was treated with PBE-GGA plus Grimme D3 dispersion^[6-7]^. Geometry optimization was carried out until the forces were converged below 0.03 eV/Å, and the self-consistent field (SCF) convergence was set at 10^-6^ Ha. Slab models of SnO_2_(110) employed ~15 Å vacuum; the bottom two layers were fixed while the top two layers and all Pd species were relaxed. Owing to the large supercells (>190 atoms), Brillouin-zone sampling used a 1×1×1 Monkhorst–Pack grid. Transition state searches were performed using the dimer method^[8-10]^ in combination with the climbing image nudged elastic band (CI-NEB) approach^[11-12]^.


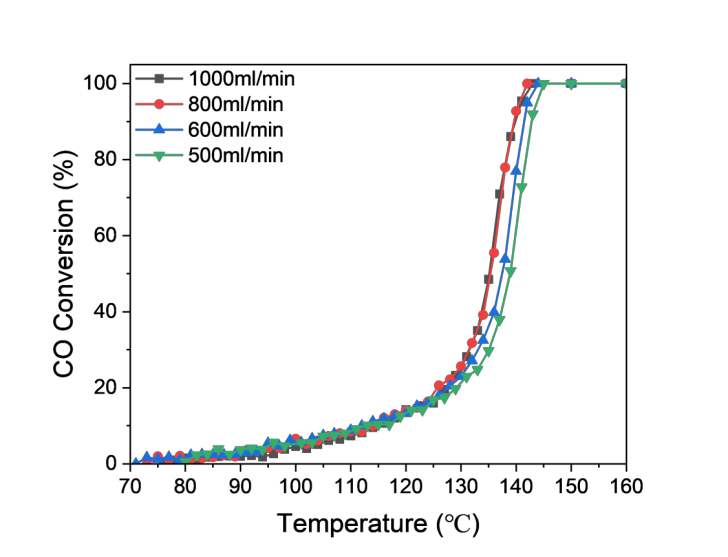


**Figure S1.** Flow test of Pd/SnO_2_-F at constant space velocity. CO 1000ppm, O_2_ 3.5%, N_2_ balance gas, total flow 500-1000mL/min, space velocity 300000 mL·h^-1^·g_cat_^-1^.

The influence of flow rate on the activity of Pd/SnO_2_-F catalyst was tested at a fixed space velocity of 300000 mL·h^-1^·g_cat_^-1^. The results showed that the activity reached its maximum when the total flow rate exceeded 800 mL/min. Therefore, a total flow rate of 1000 mL/min was selected for the activity test in this work.


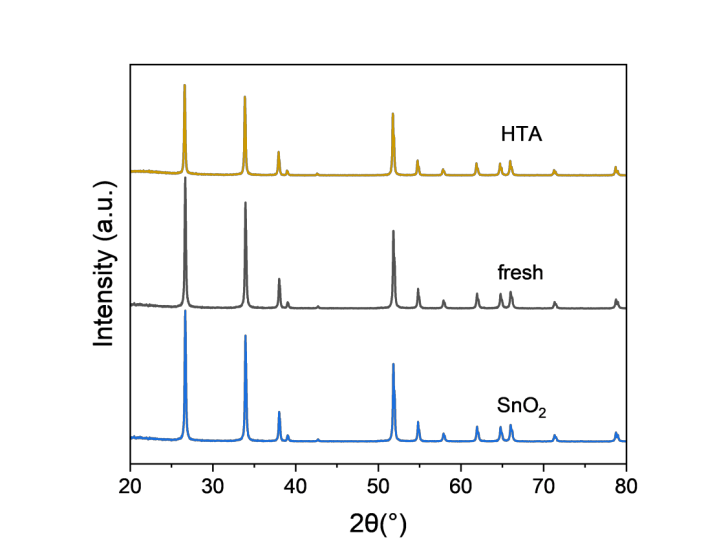


**Figure S2.** XRD patterns of SnO_2_, Pd/SnO_2_-F, and Pd/SnO_2_-H.





**Figure S3.** N_2_ adsorption-desorption isotherms of Pd/SnO_2_-F catalyst.



**Figure S4.** N_2_ adsorption-desorption isotherms of Pd/SnO_2_-H catalyst.


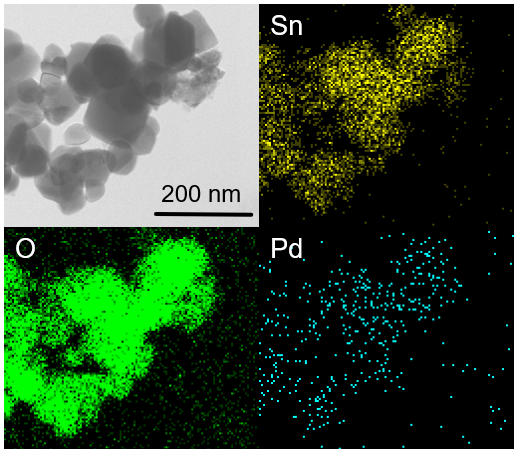


**Figure S5.** EDS mapping images of Pd/SnO_2_-H.


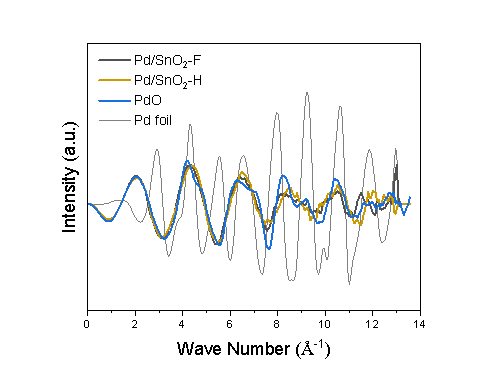


**Figure S6.** Pd K-edge *k*^2^-weighted K-space EXAFS spectra for Pd/SnO_2_-F and Pd/SnO_2_-H.





**Figure S7.** Fitting results of the k^2^ weighted R-space Pd K-edge EXAFS for Pd foil.





**Figure S8.** Fitting results of the k^2^ weighted R-space Pd K-edge EXAFS for Pd/SnO_2_-F.





**Figure S9.** Fitting results of the k^2^ weighted R-space Pd K-edge EXAFS for Pd/SnO_2_-H.


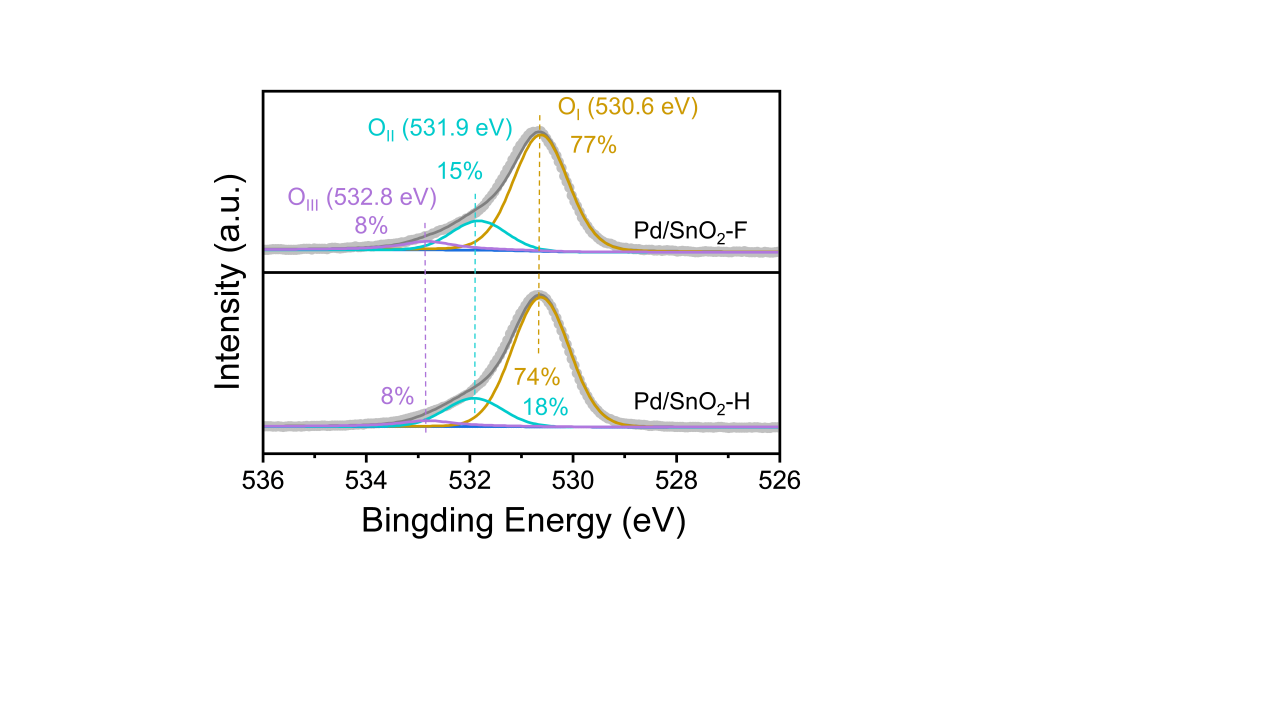


**Figure S10.** O 1s XPS profiles of Pd/SnO_2_-F and Pd/SnO_2_-H.





**Figure S11.** O_2_-TPD profiles of Pd/SnO_2_-F and Pd/SnO_2_-H catalysts.


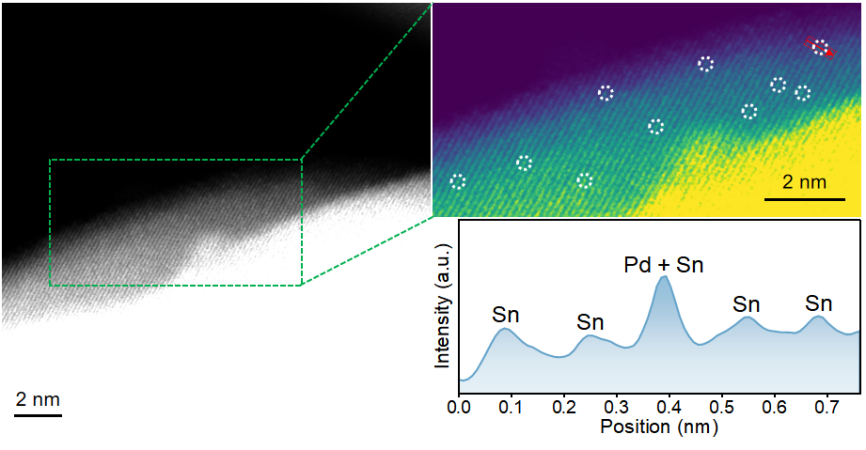


**Figure S12.** Atomic-resolution HAADF-STEM images of Pd/SnO_2_-H after a 50 h of long-term stability test. The yellow arrows and circles highlight the Pd clusters and single atoms in the false-colored image. The intensity profile is collected along the red dash-line region.


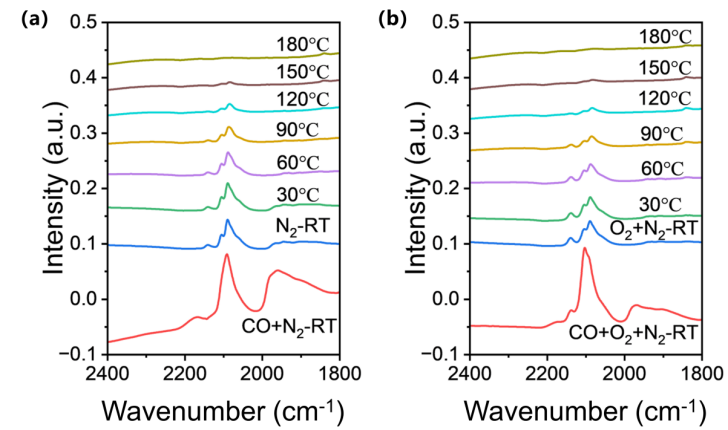


**Figure S13.** *In situ* DRIFT spectra during (a) CO-TPD and (b) CO-TPO processes over Pd/SnO_2_-H.


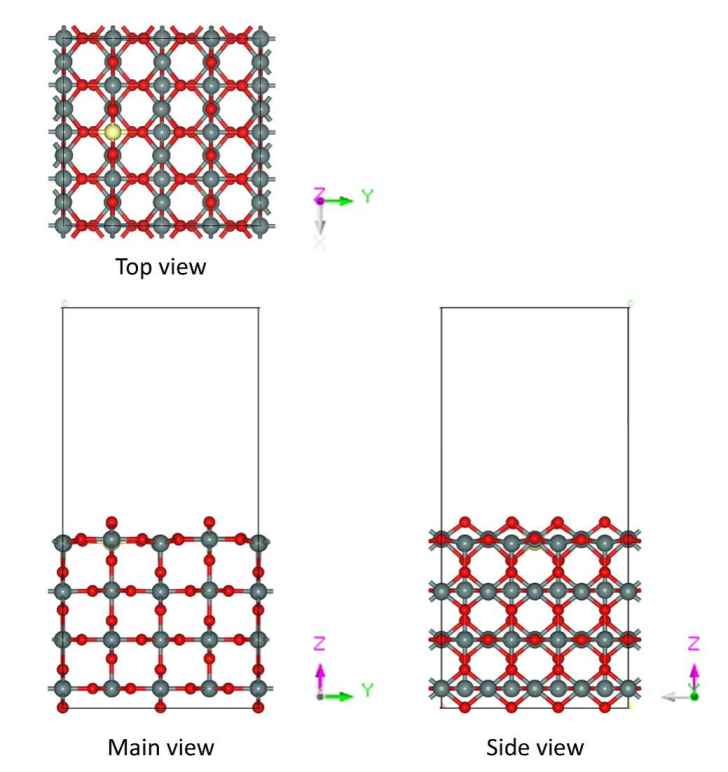


**Figure S14.** The optimized slab model of Pd_1_-SnO_2_ model.


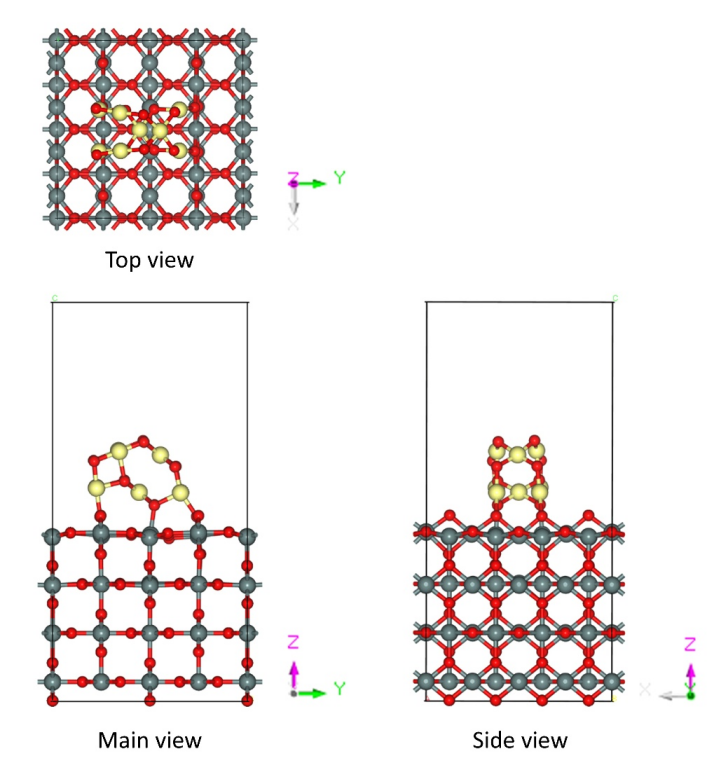


**Figure S15.** The optimized slab model of Pd_cluster_-SnO_2_ model.


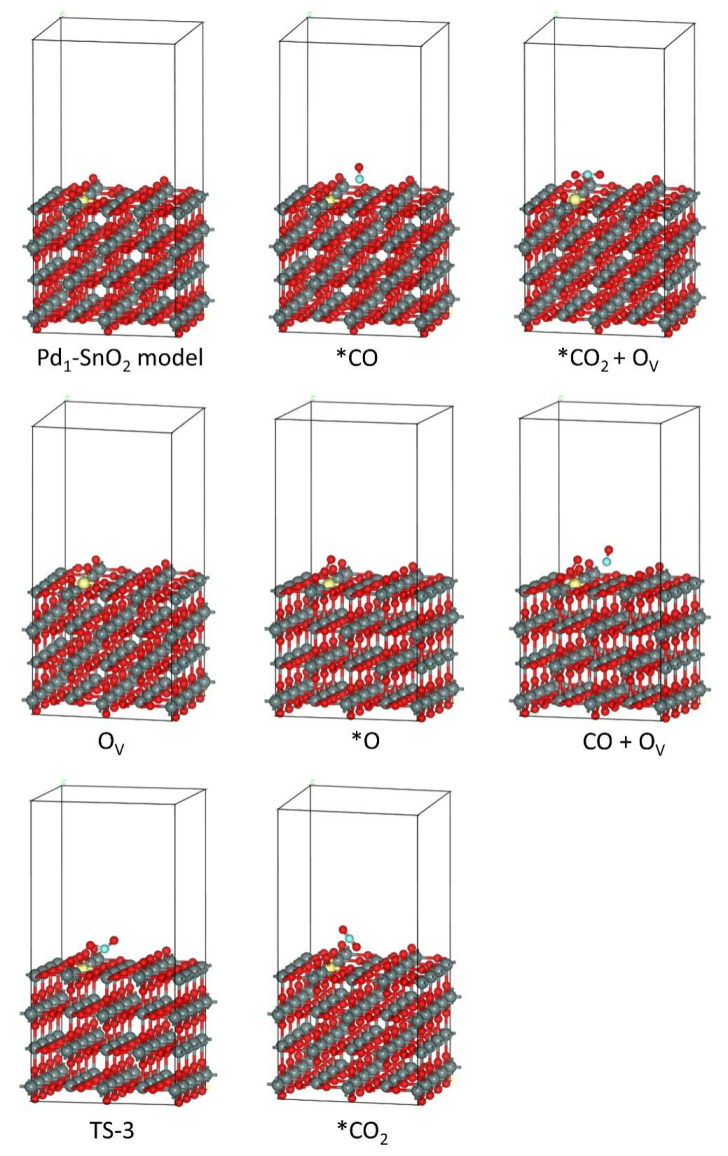


**Figure S16.** The steady-state and transition-state structures during the CO oxidation catalytic pathway on the Pd_1_-SnO_2_ model.


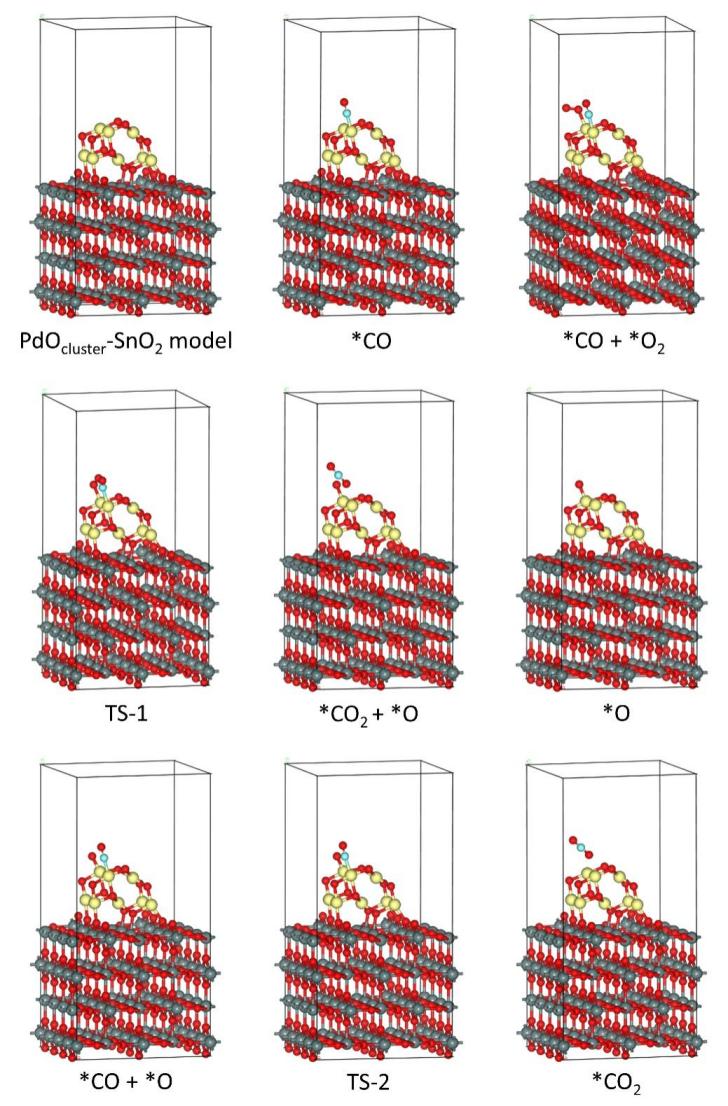


**Figure S17.** The steady-state and transition-state structures during the CO oxidation catalytic pathway on the Pd_cluster_-SnO_2_ model.


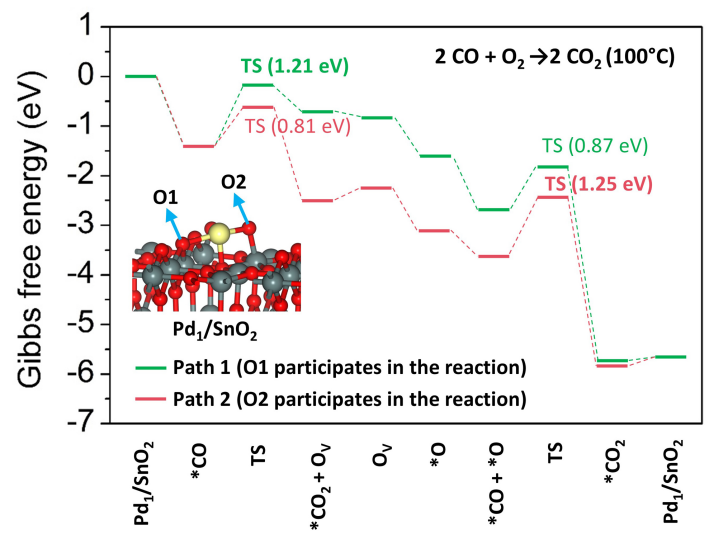


**Figure S18.** DFT-calculated Gibbs free energy profiles for two CO oxidation pathways over Pd_1_/SnO_2_ models. The Gibbs free energy values were calculated using a temperature of 100 °C.


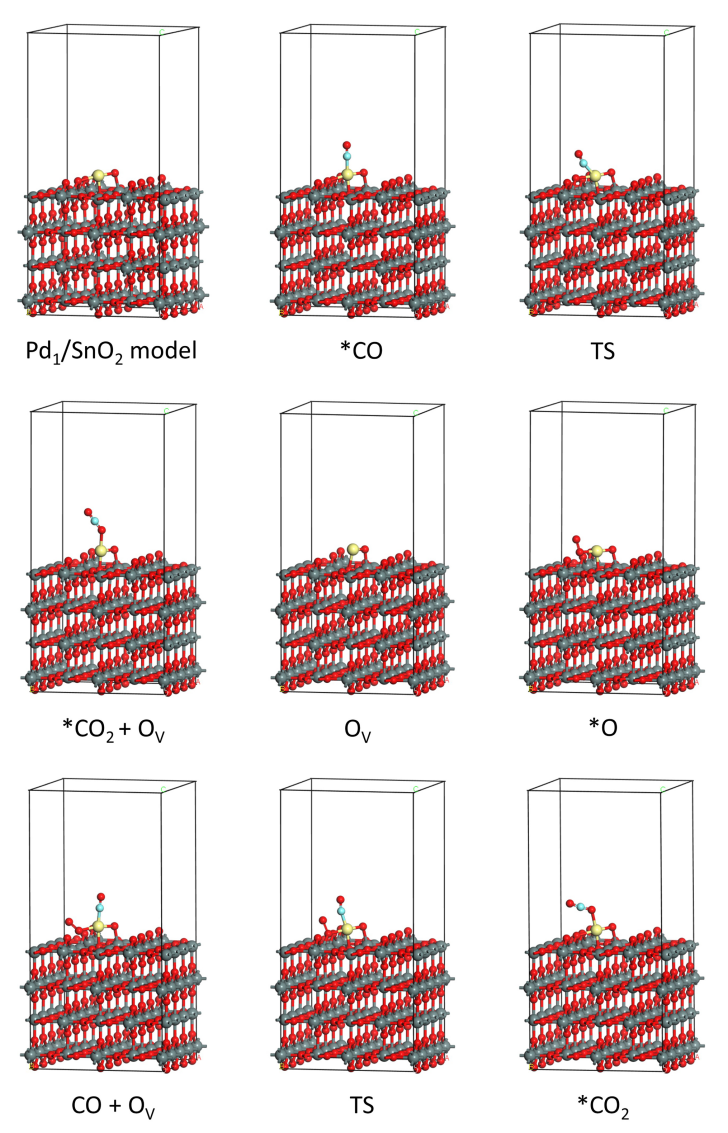


**Figure S19.** The steady-state and transition-state structures during the CO oxidation catalytic pathway (Path 1) on the Pd_1_/SnO_2_ model.


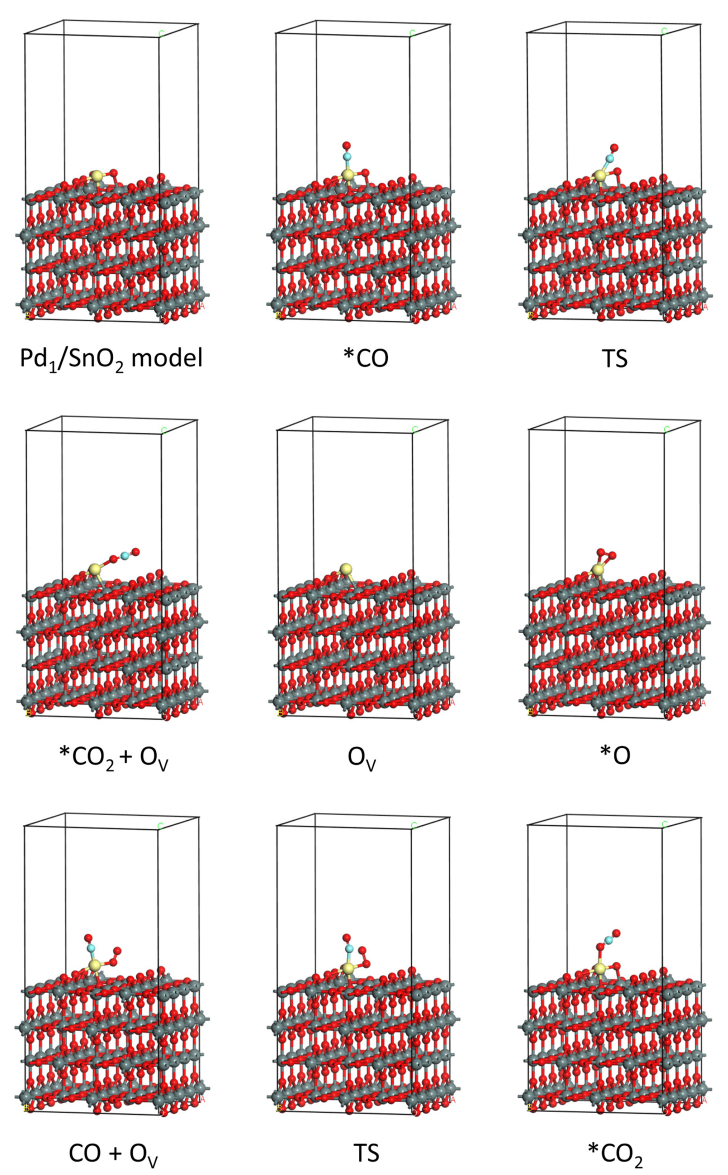


**Figure S20.** The steady-state and transition-state structures during the CO oxidation catalytic pathway (Path 2) on the Pd_1_/SnO_2_ model.

**Table S1.** BET-specific surface area of Pd/SnO_2_-F and Pd/SnO_2_-H.

| Sample | Specific surface area (m^2^/g) | Pore size (nm) | Pore volume (cm^3^/g) | Actual loading (%)^[a]^ |
| --- | --- | --- | --- | --- |
| Pd/SnO_2_-F | 8.6 | 1.28 | 0.15 | 0.15 |
| Pd/SnO_2_-H | 7.0 | 1.29 | 0.15 | 0.15 |

1. Determined by ICP-OES.

**Table S2**. List of catalyst activity in literature reports.

| Sample | Noble metal loading (%) | T_10_ (°C) | T_50_ (°C) | Reaction rate (min^-1^) | Conditions | Reference |
| --- | --- | --- | --- | --- | --- | --- |
| Pt/CeO_2__S | 1.0 | 94 | 130 | 0.58 (90 °C) | 0.4% CO，10% O_2_; 60000 mL h^−1^g_cat_^−1^ | [13] |
| Pt_1_-Ce/CeO_2-x_ | 0.044 | 130 | 180 | 4.32 (130 °C) | 1% CO, 4% O_2_; 36000 mL h^−1^g_cat_^−1^ | [14] |
| Pt-NP/CeO_2_ | 0.94 | 80 | 93 | 0.85 (100 °C) | 0.1% CO, 8% O_2_; 60000 mL h^−1^g_cat_^−1^ | [15] |
| Pd/FER | 1.8 | 70 | 110 | 0.1 (70 °C) | 0.11% CO, 15% O_2_; 2.8% H2O; 225000 mL h^−1^g_cat_^−1^ | [16] |
| Pd/CeO_2_SACs | 1.00 | 60 | 110 | 0.43 (75 °C) | 1% CO, 1% O_2_; 60000 mL h^−1^g_cat_^−1^ | [17] |
| Pd/SnO_2_-H | 0.15 | 80 | 119 | 1.44 (80 °C) | 0.1% CO, 3.5% O_2_; 300000 mL h^−1^g_cat_^−1^ | This work |

**Table S3.** Peak fitting of Pd 3*d* and O 1*s* photoelectron spectra of Pd/SnO_2_-F and Pd/SnO_2_-H.

| Sample | Pd^δ+^ (%) | Pd^2+^ (%) | Pd^4+^ (%) | O_I_ (%) | O_II_ (%) | O_III_ (%) |
| --- | --- | --- | --- | --- | --- | --- |
| Pd/SnO_2_-F | 9.5 | 90.5 | 0 | 77 | 15 | 8 |
| Pd/SnO_2_-H | 11.5 | 64.9 | 23.6 | 74 | 18 | 8 |

**Table S4.** EXAFS fitting results of Pd/SnO_2_-F and Pd/SnO_2_-H.

| Sample | Type | CN | R(Å) | σ^2^(×10^-3^Å^2^) | ΔE(eV) | R-factor |
| --- | --- | --- | --- | --- | --- | --- |
| Pd foil | Pd-Pd | 12 | 2.74 | 5.2 | -5.4 | 0.243% |
| Pd/SnO_2_-F | Pd-O | 3.44 ± 0.89 | 2.01 ± 0.03 | 2.81 ± 3.31 | -1.57 ± 3.73 | 1.14% |
| Pd/SnO_2_-H | Pd-O | 3.63 ± 0.47 | 2.03 ± 0.01 | 2.54 ± 1.61 | -1.76 ± 1.79 | 2.10% |

[1] B. Ravel, M. Newville, "ATHENA, ARTEMIS, HEPHAESTUS: data analysis for X-ray absorption spectroscopy using IFEFFIT" *J. Synchrotron Radiat.* **2005**, *12*, 537-541.

[2] G. Kresse, J. Furthmuller, "Efficient iterative schemes for ab initio total-energy calculations using a plane-wave basis set" *Phys. Rev. B*. **1996**, *54*, 11169-11186.

[3] G. K. av, J. Furthmiiller, "Efficiency of ab-initio total energy calculations for metals and semiconductors using a plane-wave basis set" *Comput. Mater. Sci.* **1996**, *6*, 15-50.

[4] P. E. Blochl, "PROJECTOR AUGMENTED-WAVE METHOD" *Phys. Rev. B*. **1994**, *50*, 17953-17979.

[5] G. Kresse, D. Joubert, "From ultrasoft pseudopotentials to the projector augmented-wave method" *Phys. Rev. B*. **1999**, *59*, 1758-1775.

[6] S. Grimme, S. Ehrlich, L. Goerigk, "Effect of the Damping Function in Dispersion Corrected Density Functional Theory" *J. Comput. Chem.* **2011**, *32*, 1456-1465.

[7] S. Grimme, J. Antony, S. Ehrlich, H. Krieg, "A consistent and accurate ab initio parametrization of density functional dispersion correction (DFT-D) for the 94 elements H-Pu" *J. Chem. Phys.* **2010**, *132*, 154104.

[8] G. Henkelman, H. Jónsson, "A dimer method for finding saddle points on high dimensional potential surfaces using only first derivatives" *J. Chem. Phys.* **1999**, *111*, 7010-7022.

[9] A. Heyden, A. T. Bell, F. J. Keil, "Efficient methods for finding transition states in chemical reactions: Comparison of improved dimer method and partitioned rational function optimization method" *J. Chem. Phys.* **2005**, *123*, 224101.

[10] J. Kaestner, P. Sherwood, "Superlinearly converging dimer method for transition state search" *J. Chem. Phys.* **2008**, *128*, 014106.

[11] G. Henkelman, B. P. Uberuaga, H. Jónsson, "A climbing image nudged elastic band method for finding saddle points and minimum energy paths" *J. Chem. Phys.* **2000**, *113*, 9901-9904.

[12] G. Henkelman, H. Jónsson, "Improved tangent estimate in the nudged elastic band method for finding minimum energy paths and saddle points" *J. Chem. Phys.* **2000**, *113*, 9978-9985.

[13] L. Nie, D. H. Mei, H. F. Xiong, B. Peng, Z. B. Ren, X. I. P. Hernandez, A. DeLariva, M. Wang, M. H. Engelhard, L. Kovarik, A. K. Datye, Y. Wang, "Activation of surface lattice oxygen in single-atom Pt/CeO_2_ for low-temperature CO oxidation" *Science*. **2017**, *358*, 1419-1423.

[14] J. Yang, L. J. Falling, S. Y. Yan, B. L. Zhang, P. Verma, L. Daemen, Y. Q. Cheng, X. Zhao, S. C. Zhang, J. L. Chen, B. Q. Yao, S. D. Tan, S. Chae, Q. He, S. Nemsak, Z. L. Wu, D. Prendergast, Y. B. Guo, J. X. Liu, M. Salmeron, J. Su, "Formation of hydrided Pt-Ce-H sites in efficient, selective oxidation catalysts" *Science*. **2025**, *388*, 514-519.

[15] F. Maurer, J. Jelic, J. J. Wang, A. Gänzler, P. Dolcet, C. Wöll, Y. M. Wang, F. Studt, M. Casapu, J. D. Grunwaldt, "Tracking the formation, fate and consequence for catalytic activity of Pt single sites on CeO_2_" *Nat. Catal.* **2020**, *3*, 824-833.

[16] I. H. Song, I. Z. Koleva, H. A. Aleksandrov, L. X. Chen, J. Heo, D. S. Li, Y. Wang, J. Szanyi, K. Khivantsev, "Ultrasmall Pd Clusters in FER Zeolite Alleviate CO Poisoning for Effective Low-Temperature Carbon Monoxide Oxidation" *J. Am. Chem. Soc.* **2023**, *145*, 27493-27499.

[17] V. Muravev, G. Spezzati, Y. Q. Su, A. Parastaev, F. K. Chiang, A. Longo, C. Escudero, N. Kosinov, E. J. M. Hensen, "Interface dynamics of Pd-CeO_2_ single-atom catalysts during CO oxidation" *Nat. Catal.* **2021**, *4*, 469-478.
